# Prevalence of Asymptomatic Gallstone in Healthy Neonates in Shiraz, Southern Iran

**Published:** 2011-11-01

**Authors:** M R Bordbar, R Karami, K Kamali, N Pishva, M Haghighat

**Affiliations:** 1 Hematology Research Center, Nemazee Hospital,Shiraz University of Medical Sciences, Shiraz, Iran; 2 Department of Pediatrics, Shiraz University of Medical Sciences, Shiraz, Iran; 3 Department of Radiology, Shiraz University of Medical Sciences, Shiraz, Iran

**Keywords:** Prevalence, Asymptomatic, Gallstone, Neonate, Iran

Dear Editor,

Gallstone is a rare but well-known finding in neonates which is diagnosed more easily nowadays. It has been associated with hematologic disorders, prematurity, prolonged fasting, parenteral nutrition, illeal resection, dehydration, and phototherapy, congenital abnormalities of the biliary tract, E. coli sepsis, furesemide therapy and pseudohypoaldosteronism.[1][2][3]As neonatal gallstones are usually asymptomatic and incidentally found during routine ultrasonography, we decided to evaluate the frequency and significance of asymptomatic neonatal gallstone in healthy neonates.

This prospective study was conducted on 761 healthy neonates (53.5% male and 92.2% term), born in a university-affiliated nursery from January 2008 through December 2009. The neonates had no medical or surgical illness and were evaluated with ultrasonography (model logic 7, General Electric Milvaki USA) by an expert radiologist during the first seven days of their life, searching for any signs of gallbladder stone or sludge. Diagnosis of gallstone was based on the presence of echogenic foci in the gallbladder lumen or acoustic shadowing. Repository of data included neonate’s birth date, weight at birth, route of delivery, gender, family history of gallstone, mother’s drug history during pregnancy as well as her medical history of the gestational diabetes and hypertension or pre-eclampsia. Follow-up sonography was repeated after 2 weeks for those discovered to have gallstones.

**Fig. 1:**
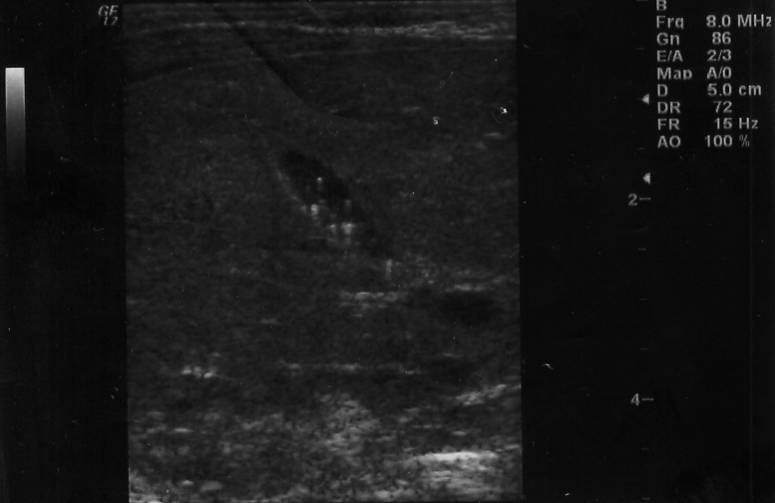
Multiple hyperechoic structures with posterior reverberation in 4 days old neonate.

**Fig. 2:**
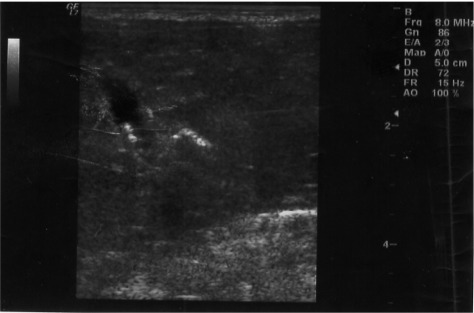
Two high echogenic stones with posterior shadowing in 3 days old neonate

About half of babies (49.7%) were delivered by cesarean section (C/S). Family history of gallstone was positive in 17 (2.2%) cases. There was no history of chronic medical diseases and the mothers only used folic acid, iron supplements and multi-vitamins during their gestation. None of the babies in our study required intensive phototherapy or exchange transfusion for their hyperbilirubinemia. Cholelithiasis was diagnosed in 2 out of 761 neonates with the prevalence of 0.26%. The first case was a 3 days term male neonate, product of C/S, with birth weight of 3200 g, normal physical exam and in good general health. His sonography revealed multiple echogenic foci in the gallbladder (Figure 1). The second case was a 2 days term female neonate who was delivered by normal vaginal delivery with birth weight of 2950 g. The sonography demonstrated three echogenic foci with acoustic shadowing in her gallbladder (Figure 2). A follow-up sonogram was performed after 2 weeks and showed spontaneous resolution of gallstones in both cases without any medical or surgical intervention.

Silent gallstones which are accidentally found during routine ultrasonography constituted a great deal of neonatal gallstones. Mostly, no etiologic factor was identified and they might have fetal origin.[1][4][5][6] The prevalence of neonatal gallstone was variable in different studies, ranging from 0.13% to 1.9%.[6][7][8][9] We found it to be about 0.26% comparable to the results of Palasciano et al. and Gilger.[7][8] Due to small number of positive cases in our study, we can not confidently report the predisposing factors of neonatal cholelithiasis. However, in contrast to most studies, neonate’s gender was not a risk factor in our study similar to Munjuluri et al.’s investigation.[4] We found no identifiable risk factors in the two neonates with gallstones, although they were too small to be analyzed.

There are many controversies regarding the best therapeutic strategy. While some authors suggested medical therapy such as ursodeoxycholic acid,[4] others recommend close observation on asymptomatic cases.[1][10] Neonatal gallstone appears to be a temporary, self-limiting phenomenon, which does not warrant any heroic or aggressive approach, unless it is associated with stricture or congenital anatomic abnormalities of the common bile duct. Cholecystectomy is reserved for complicated cases. It seems that observation and follow-up sonograms are the only objective requirements to perform.
